# Spirometry is not enough to diagnose COPD in epidemiological studies: a follow-up study

**DOI:** 10.1038/s41533-017-0062-6

**Published:** 2017-11-14

**Authors:** Elena Andreeva, Marina Pokhaznikova, Anatoly Lebedev, Irina Moiseeva, Olga Kuznetsova, Jean-Marie Degryse

**Affiliations:** 1 Institute of Health and Society, Université Catholique de Louvain, IRSS, Clos Chapelle-aux-Champs, 30/10.15, 1200 Brussels, Belgium; 2 Department of Family Medicine, Northern State Medical University, pr. Troitsky, 51, 163000 Arkhangelsk, Russia; 3 Department of Family Medicine, North-Western State Medical University named after I.I. Mechnikov, Kirochnaya str., 41, 191015 St. Petersburg, Russia; 4 Department of Public Health and Primary Health Care, K.U.Leuven, Kapucijnenvoer 33, B3000 Leuven, Belgium

## Abstract

A hallmark of the diagnosis of chronic obstructive pulmonary disease (COPD) is the measurement of post-bronchodilator (post-BD) airflow obstruction (AO) by spirometry, but spirometry is not enough for the provision of a clinical diagnosis. In the majority of previous epidemiological studies, COPD diagnosis has been based on spirometry and a few clinical characteristics. The aim of our study was to identify outcomes in patients newly diagnosed with airflow obstruction (AO) based on a diagnostic work-up conducted as part of a population-based cross-sectional study in North-Western Russia. Spirometry was performed before (pre-BD) and after BD administration, and AO was defined using the FEV1/FVC <0.70 and FEV1/FVC <lower limit of normal cut-off values. Relevant symptoms were recorded. Participants with AO identified at baseline were then examined by a pulmonologist, including a clinical examination and second spirometry with BD test. Of the 102 participants with post-BD AO in the initial assessment, only 60.8% still had AO identified at the second examination; among these patients, the following final diagnoses were reported: COPD (*n* = 41), asthma (*n* = 5), asthma–COPD overlap syndrome (ACOS) (*n* = 4) and likely ACOS (*n* = 5). Of the 65 participants with pre-BD AO, 23.1% had post-BD AO at the second assessment, and these patients had been diagnosed with COPD (*n* = 12), asthma (*n* = 1), ACOS (*n* = 1), likely ACOS (*n* = 1). Serial spirometric assessments complemented by a comprehensive clinical evaluation are recommended in new epidemiological studies.

## COPD: Single lung function test not enough for diagnosis

A single lung function test is insufficient to accurately diagnose chronic obstructive pulmonary disease (COPD). A team led by Jean-Marie Degryse from the Université Catholique de Louvain, Belgium, studied patients in Northwestern Russia who showed signs of airflow obstruction, as measured by a spirometer, a medical device that records the amount of air inhaled and exhaled and the speed of breath. Among those who initially showed signs of limited lung function, even after treatment with a bronchodilator, a large proportion did not exhibit airflow obstruction on a second spirometry test months later. Moreover, not everyone who had confirmed airflow obstruction were diagnosed with COPD when examined by a pulmonologist. Some had asthma or asthma-COPD overlap syndrome. The findings point to the need for clinical assessments and follow-up spirometry tests to accurately diagnose and manage COPD.

## Introduction

Although chronic obstructive pulmonary disease (COPD) is, at present, the third most prevalent cause of death in developed countries and associated with increasing mortality in developing countries, this conditions is still not well recognized by either the general public or physicians, and over half of COPD cases go undiagnosed.^1^ The rates of COPD prevalence, morbidity, and mortality vary across countries and different groups within countries.^2^ According to the Burden of Obstructive Lung Disease study, the prevalence of stage II or higher COPD was 10.1% overall (from 5.9% in Germany to 19.1% in South Africa), 11.8% in men, and 8.5% in women, mainly increasing with age and smoking exposure.^3^

In 2011, the European Respiratory Society (ERS) task force report entitled “Recommendations for Epidemiological Studies of COPD” was published with the aim of establishing clear diagnostic criteria and standardized methods to examine COPD.^4^ The authors strongly recommended measuring as many different characteristics of COPD patients (e.g., respiratory symptoms, exacerbation frequency, comorbidity assessment, body mass index (BMI), biological markers, chest radiography, and risk factors assessment) as possible, in addition to spirometry, to provide a better understanding of the disease.^4^

There are several different problems related to the diagnosis of COPD. The lack of consensus on its definition should be given primary consideration. Several definitions coexist, but no one definition is preferred over the others.^5^ COPD has been defined as a complex and heterogeneous syndrome with pulmonary and extrapulmonary features;^2^ as a variety of different clinical syndromes based on the presence of symptoms, measures of airflow obstruction (AO) and reversibility;^5,6^ or as a heterogeneous collection of diseases with different causes, pathogenic mechanisms, and physiological effects.^7^ The guidelines of the Global Initiative of Chronic Obstructive Lung Disease (GOLD) defined COPD in 2016 as “a common, preventable and treatable disease characterized by persistent airflow limitation that is usually progressive, and associated with an enhanced chronic inflammatory response in the airways and the lungs to noxious particles or gases. Exacerbations and comorbidities contribute to the overall severity in individual patients”.^2^

Another problem is the use of different COPD definitions in epidemiological and case-finding studies. Moreover, COPD definitions within each type of study have also varied. As recommended by several recent international guidelines or research initiatives, standardization of the epidemiological definition of COPD is one of the key elements necessary for the estimation of COPD prevalence.^8^ There is significant heterogeneity in the estimation of COPD prevalence, even in well-designed epidemiologic studies, due to the diverse methodological approaches applied to COPD definition and diagnostics, investigation methods used, and target populations studied.^8,9^ Even when using data from the same study, different definitions and exclusion criteria can result in different estimates of COPD prevalence.^9^ A similar problem can be observed in studies evaluating case-finding strategies for COPD.^10^ A standardized epidemiological definition of COPD is needed to conduct high-quality randomized controlled trials designed to compare target populations, recruitment strategies, and screening tests.^11^

The third issue is the absence of a gold standard AO cut-off value. The two most frequently used values are as follows: a fixed ratio of 0.7 (a ratio of the forced expiratory volume in 1 s to the forced vital capacity, FEV1/FVC <0.7) and a FEV1/FVC ratio below the fifth percentile of a large healthy reference group (the statistically defined lower limit of normal, LLN).^2^ This case-finding strategy assumes that spirometry has been performed on patients with risk factors and respiratory symptoms and that if AO (with fixed ratio FEV1/FVC <0.70) is identified, a diagnosis of COPD will be confirmed unless patients have other respiratory diseases, such as asthma, bronchiectasis, or stenosing bronchial tumors.^5^

The LLN cut-off has been the preferred measure for use in epidemiological settings, as it is the physiologists’ choice and uses a definition based on normality.^4,5^ In the clinical setting, the fixed cut-off is more simple and familiar, but it is difficult to choose one appropriate cut-off because of absence of comparative studies.^5^ Using the fixed cut-off, the COPD prevalence is often higher than it is when estimated using the LLN cut-off.^9^ Sex differences in the risk of COPD are also influenced when the fixed cut-off definition is used for diagnosing COPD; no such difference has been found when using the LLN definition.^12^

The next important issue in COPD diagnosis is the uncertainty of diagnosis over time, as an individual can be diagnosed with mild COPD at first assessment but have normal spirometry results at follow-up, even without intervention.^13^ Both the forced expiratory volume in one second (FEV1) and the forced vital capacity (FVC) can vary over time.^13^ There is still room for improvement in the diagnostic work-up of COPD, such as defining a cut-off value using a ratio of the FEV1 to the forced expiratory volume in 6 s (FEV1/FEV6) <LLN, defining a borderline zone around the LLN, or repeating spirometry for patients with borderline results.^13^

The ERS task force also recommends the assessment of various COPD phenotypes.^4^ One of these phenotypes is asthma–COPD overlap syndrome (ACOS), which was recently presented in the Global Strategy for Diagnosis, Management and Prevention of COPD (GOLD).^2^ ACOS is characterized by clinical features common to both asthma and COPD, which makes the diagnosis of COPD even more challenging.^2^ The characterization of ACOS, which currently has different definitions, remains preliminary, which might lead to heterogeneous estimates of ACOS prevalence.^14^ This is aggravated by the fact that both asthma and COPD are heterogeneous diseases with substantial inter-individual variability, and varied pathogenic mechanisms and risk factors.^15^ In addition, large population studies have found that a high proportion of patients with respiratory problems may be classified as having more than one diagnosis.^9^

To summarize, the key issues regarding the diagnosis of COPD include different COPD definitions, a gap between the “epidemiological” definition and the “clinical definition” of COPD, two proposed FEV1/FVC cut-off values for defining AO, and uncertainty in the diagnosis over time and as it relates to ACOS. Further studies are needed to combine the epidemiological and clinical perspectives and, thus, to improve the diagnosis of COPD.

Recently, we reported for the first time the prevalence of AO in adults aged 35–70 years in two northwestern cities in Russia using both the fixed and LLN (by the Global Lung Initiative (GLI) 2012 reference equations) cut-off values; additionally, we identified risk factors and assessed the diagnostic value of respiratory symptoms for AO (“The RESearch on the PrEvalence and diagnosis of COPD and its Tobacco-related etiology”, RESPECT study).^16,17^ This article reports the findings of a follow-up diagnostic assessment performed by a pulmonologist on those identified as having AO during the baseline assessment of the RESPECT study.

## Results

**Fig. 1:**
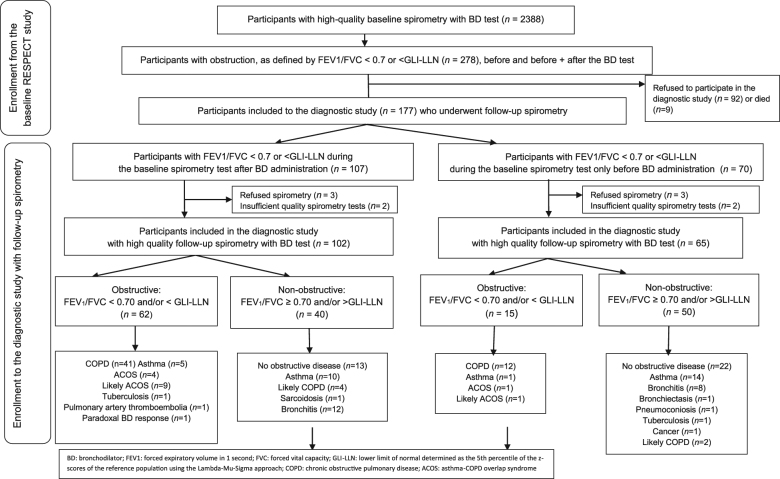
The RESPECT Diagnostic Study Flow Diagram

Of the 3133 individuals included in the RESPECT study, 2974 had satisfactory pre-BD and 2388 had satisfactory post-BD spirometry. All 278 individuals with pre-BD and pre + post-BD AO based on the fixed or GLI–LLN cut-off values were invited to participate in this diagnostic study; of these patients, 177 agreed to participate and 167 had satisfactory post-BD spirometry (Fig. 1). The mean interval between the baseline and the second spirometry tests was 14.5 ± 4.8 months (ranging from 4 to 27 months).

The mean age of the participants was 56.6 years; of the participants, 51.5% were male and 74.3% were current or ex-smokers. The baseline characteristics of the study population, including BD test results and seven diagnostic categories based on clinical and spirometric criteria, are presented in Table 1.

**Table 1:** Baseline characteristics of the total population across diagnosis categories

	^e^Total population, *n* = 167	^f^COPD, *n* = 53	^g^Likely COPD, *n* = 6	Asthma, *n* = 30	^h^ACOS and likely ACOS, *n* = 15	^i^Chronic bronchitis, *n* = 20	^j^Other diagnosis, *n* = 8	No respiratory diseases, *n* = 35
Age (years), mean ± SD	56.6 ± 8.50	58.5 ± 7.51	58.5 ± 7.34	56.4 ± 8.42	60.6 ± 4.34	53.1 ± 9.48	52.0 ± 9.65	55.0 ± 9.64
Men, *n* (%)	86 (51.5)	42 (79.2)	6 (100)	4 (13.3)	3 (20)	11 (55.0)	3 (37.5)	17 (48.6)
Smoking status	166	53	6	29	9	20	8	35
Smoker/ex-smoker, *n* (%)	124 (74.3)	50 (94.3)	6 (100)	11 (36.7)	9 (60.0)	17 (85.0)	7 (87.5)	24 (68.6)
Smoking exposure^a^, *n* (%)	165	52	6	29	15	20	8	35
Never smoker	43 (26.1)	3 (5.8)	-	19 (65.5)	6 (40.0)	3 (15.0)	1 (12.5)	11 (31.4)
<10 pack-years	25 (15.2)	4 (7.7)	1 (16.7)	4 (13.8)	3 (20.0)	2 (10.0)	3 (37.5)	8 (22.9)
≥10 and <20 pack-years	19 (11.5)	5 (9.6)	5 (83.3)	2 (6.9)	3 (20.0)	4 (20.0)	1 (12.5)	4 (11.4)
≥20 pack-years	78 (47.3)	40 (76.9)	-	4 (13.8)	3 (20.0)	11 (55.0)	3 (37.5)	12 (34.3)
Dusty job^b^	162	52	6	29	14	20	7	34
≥10 years, *n* (%)	43 (25.7)	13 (24.5)	1 (16.7)	6 (20.0)	3 (21.4)	5 (25.0)	2 (28.6)	13 (38.2)
Gas (fumes) job^c^	162	52	6	29	14	20	7	34
≥10 years, *n* (%)	47 (28.1)	16 (30.2)	1 (16.7)	5 (17.2)	4 (28.6)	5 (20.0)	2 (28.6)	14 (41.2)
Symptoms^d^	113 (67.7)	37 (69.8)	6 (100)	19 (63.3)	13 (86.7)	13 (65.0)	5 (65.5)	20 (57.1)
Current respiratory disease (reported by patient)	164	52	-	29	15	20	8	34
Asthma, *n* (%)	31 (18.6)	3 (5.7)	-	19 (65.5)	9 (60.0)	-	-	-
Any allergic diseases, *n* (%)	43 (25.7)	5 (9.4)	-	19 (65.5)	6 (40.0)	4 (20.0)	1 (12.5)	8 (23.5)
Chronic bronchitis, *n* (%)	39 (23.4)	19 (35.8)	-	7 (24.1)	5 (33.3)	3 (15.0)	-	2 (5.9)
Emphysema, *n* (%)	2 (1.2)	1 (1.9)	-	-	1 (6.7)	-	-	-
COPD, *n* (%)	17 (10.2)	9 (17.0)	-	4 (13.8)	6 (40.0)	-	-	-
No respiratory diseases, *n* (%)	100 (59.9)	28 (52.8)	6 (100)	7 (24.1)	5 (33.3)	17 (85.0)	5 (62.5)	32 (94.1)
Family history of asthma, *n* (%)	20 (12.0)	4 (7.5)	-	8 (27.6)	1 (6.7)	1 (5.0)	1 (12.5)	5 (14.3)
Family history of chronic bronchitis, *n* (%)	18 (10.8)	6 (11.3)	1 (16.7)	4 (13.8)	2 (13.3)	1 (5.0)	1 (12.5)	3 (8.6)
FEV1 (L), mean ± SD	2.51 ± 0.81	2.35 ± 0.81	2.85 ± 0.76	2.35 ± 0.68	1.72 ± 0.43	3.03 ± 0.64	2.54 ± 1.22	2.88 ± 0.71
FVC (L), mean ± SD	3.63 ± 1.03	3.84 ± 1.01	3.86 ± 1.02	3.17 ± 0.84	2.70 ± 0.75	3.98 ± 0.87	3.63 ± 1.66	3.83 ± 0.10
FEV1/FVC, mean ± SD	0.69 ± 0.10	0.60 ± 0.10	0.73 ± 0.06	0.73 ± 0.06	0.62 ± 0.06	0.76 ± 0.03	0.71 ± 0.10	0.75 ± 0.05

*FEV1* forced expiratory volume in 1 s, *FVC* forced vital capacity, *GLI–LLN* lower limit of normal, determined as the 5th percentile of the z-scores of the reference population using the Lambda-Mu-Sigma approach

^a^ One pack-year of smoking indicates that an individual smoked one package of cigarettes (20 cigarettes) daily for 1 year

^b^ Working at a dusty job for more than 1 year

^c^ Working at a gas/fumes job more than 1 year

^d^ Chronic respiratory symptoms were defined as the presence of chronic cough and chronic phlegm (on most days for at least 3 months each year) and chronic dyspnea

^e^ Participants included in the diagnostic study (with pre and/or post obstruction indicated by FEV1/FVC < 0.7 or FEV1/FVC<GLI–LLN

^f^ Including participants with clear chronic obstructive pulmonary disease (COPD) = 46, COPD with tuberculosis = 1, COPD with pneumoconiosis = 3, COPD with cancer = 2; COPD with systemic disease = 1

^g^ Participants with symptoms and risk factors of COPD but no observed AO (FEV1/FVC > 0.7 or FEV1/FVC>GLI–LLN)

^h^ Asthma–COPD overlap syndrome (ACOS): Participants with features that are common to both asthma and COPD and with post-BD FEV1/FVC < 0.7 or FEV1/FVC<GLI–LLN and post-BD increase in FEV1 > 12% and 400 ml from baseline; likely ACOS: Participants with features that are common to both asthma and COPD and with post-BD FEV1/FVC < 0.7 or FEV1/FVC<GLI–LLN and post-BD increase in FEV1 > 12% and 200 ml from baseline

^i^ Participants with a productive cough lasting for at least 3 months and with recurring bouts occurring for at least two consecutive years

^j^ Including participants with tuberculosis (*n* = 2), sarcoidosis (*n* = 1), pneumoconiosis (*n* = 1), bronchiectasis (*n* = 1), cancer (*n* = ), pulmonary artery thromboembolia (*n* = 1), negative BD response (*n* = 1)

There was no significant difference between the health statuses of participants who had AO identified during the follow-up spirometry measurement and those who did not. Overall, 37.3% of those who had and 27.6% of those who did not have AO reported that their health status had worsened during the last year (*p* = 0.23).

Of the 167 participants with AO observed during the first spirometry examination, 46.1% had post-BD AO identified during the second spirometry examination (23.1% of those with pre-BD AO and 60.8% of those with post-BD AO during the first spirometry examination) (Fig. 1). The participants with pre-BD and post-BD AO identified based on both FEV1/FVC cut-offs had mainly been diagnosed as having COPD (68.8%), ACOS or likely ACOS (19.5%), and asthma with fixed obstruction (7.8%) (Fig. 1). Among those without AO identified during the second spirometry examination, 35 participants (38.9%) had been diagnosed with no objective evidence of obstructive lung disease. The other main diagnostic categories were asthma (26.7%) and chronic bronchitis (22.2%). The participants with post-BD AO tended to more often be male (61%), older (mean age 58.6 ± 6.84 years) and smokers/ex-smokers (83.1%) compared to participants without AO. There were no statistically significant differences between the AO categories regarding occupational hazards (dusty or gas/chemical fumes exposure for more than 10 years). Forty-seven percent of the participants with AO did not report a diagnosis of any chronic respiratory disease, and 26% of those with AO did not report any chronic respiratory symptoms.

The FEV1/FVC ratio measured during the baseline spirometry was lower among the 62 participants who had post-BD AO identified both during the first and second spirometry measurements than among the 40 participants without AO identified during the second spirometry measurement (FEV1/FVC = 0.60 [95% CI 0.57–0.65) and FEV1/FVC = 0.66 [95% CI 0.65–0.67], correspondently, *p* < 0.01). These 62 participants were older than the other 40 participants (mean ages of 59.5 ± 6.6 and 56.3 ± 8.8 years reported at baseline spirometry, respectively, *p* = 0.000) and tended to be male (61.3 vs. 50%, *p* = 0.32).

The positive predictive values (PPVs) of the syndrome including all the chronic respiratory symptoms and of chronic cough and dyspnea separately was low for the diagnosis of COPD (and “likely COPD”), asthma, ACOS and chronic bronchitis (Table 2).

**Table 2:** Predictive value of chronic respiratory symptoms for chronic respiratory diseases

	COPD and likely COPD (*n* = 53 + 6)		Asthma (*n* = 30)		ACOS and likely ACOS (*n* = 5 + 15)		Chronic bronchitis (*n* = 20)	
	Positive	Negative	Positive	Negative	Positive	Negative	Positive	Negative
Chronic respiratory symptoms^a^								
With symptoms (*n* = 113)	43	70	19	94	13	100	13	100
No symptoms (n = 54)	16	38	11	43	2	52	7	47
Total (*n* = 167)	59	108	30	137	15	152	20	147
Sensitivity, % (95% CI)	72.88 (59.73–83.64)		63.33 (43.86–80.07)		86.67 (55.54–98.38)		65.00 (40.78–84.61)	
Specificity, % (95% CI)	35.19 (26.24–44.96)		31.39 (23.73–39.87)		34.21 (26.72–43.33)		31.97 (24.53–40.16)	
AUC (95% CI)	0.54 (0.45–0.63)		0.47 (0.35–0.58)		0.61 (0.47–0.74)		0.49 (0.35–0.62)	
Positive predictive value, % (95% CI)	38.05 (29.08–47.67)		16.81 (10.44–25.01)		11.50 (6.27–18.07)		11.50 (6.27–18.87)	
Negative predictive value, % (95% CI)	70.37 (56.39–82.02)		79. 63 (66.47–89.37)		96.30 (87.25–99.55)		87.04 (75.10–94.63)	
Chronic cough^b^								
With cough (*n* = 98)	40	58	16	82	9	89	10	88
No cough (*n* = 69)	19	50	14	55	6	63	10	59
Total (*n* = 167)	59	108	30	137	15	152	20	147
Sensitivity, % (95% CI)	67.80 (54.36–79.38)		55.17 (35.69–73.55)		60.00 (32.29–83.66)		50.00 (27.20–72.80)	
Specificity, % (95% CI)	45.79 (36.12–55.70)		40.15 (31.87–48.86)		41.06 (33.13–49.35)		39.73 (31.73–48.15)	
AUC (95% CI)	0.57 (0.48–0.66)		0.48 (0.36–0.59)		0.51 (0.35–0.66)		0.45 (0.31–0.58)	
Positive predictive value, % (95% CI)	40.82 (30.99–57.21)		16.33 (9.63–25.16)		9.18 (4.29–16.72)		10.20 (5.00–17.97)	
Negative predictive value, % (95% CI)	72.46 (60.38–82.27)		80.88 (69.53–89.41)		91.18 (81.78–96.69)		85.29 (74.61–92.72)	
Chronic dyspnea^c^								
With dyspnea (*n* = 85)	31	54	16	69	12	73	8	77
No dyspnea (*n* = 82)	28	54	14	68	3	79	12	70
Total (*n* = 167)	59	108	30	137	15	152	20	147
Sensitivity, % (95% CI)	52.54 (39.12–65.70)		55.17 (35.69–73.55)		80.00 (51.91–95.67)		40.00 (19.12–63.95)	
Specificity, % (95% CI)	50.00 (40.22–58.78)		49.64 (40.99–58.30)		51.66 (43.39–59.85)		47.26 (38.95–55.68)	
AUC (95% CI)	0.51 (0.42–0.60)		0.52 (0.41–0.64)		0.66 (0.53–0.79)		0.44 (0.30–0.57)	
Positive predictive value, % (95% CI)	36.47 (26.29–47.62)		18.82 (11.16–28.76)		14.12 (7.51–23.36)		9.41 (4.15–17.71)	
Negative predictive value, % (95% CI)	65.85 (54.55–75.97)		83.95 (74.12–91.17)		96.30 (89.56–99.23)		85.19 (75.55–92.10)	

The assessment of the diagnostic value of signs and symptoms performed in our analysis might be affected by inclusion bias, since the evaluation of those symptoms is part of the final diagnosis (=reference standard). Nevertheless we think that our analysis might reflect the way signs and symptoms are used in clinical practice

*COPD* chronic obstructive pulmonary disease, *ACOS* asthma–COPD overlap syndrome, *CI* confidence interval

^a^ Chronic respiratory symptoms were defined as the presence of chronic cough and chronic phlegm on most days for at least 3 months each year and chronic dyspnea

^b^ Chronic cough was defined as the presence of any cough first thing in the morning or during the day or on most days for at least 3 months each year

^c^ Chronic dyspnea was defined as the presence of any dyspnea (shortness of breath when hurrying on level ground or walking up a slight hill or walking with other people of his/her own age on level ground or having to stop for breath when walking at his/her pace on level ground).

We tabulated participants with a diagnosis of AO at baseline by GOLD class in order to compare how many changed diagnosis after the second assessment in each class (supplementary table 1). We found that 60% of those that shifted diagnosis from obstructive to non-obstructive were labeled as GOLD class 1 before and another 37,5% as GOLD class 2

In order to identify which patients would require serial spirometry, we compared the background characteristics at baseline of two subgroups: those that remained obstructed after the second assessment and those that presented a shift from obstructive to non-obstructive (see the supplementary table 2). We found that those who shifted from non-obstructive were older had lower FEV1 and FEV1/FVC values, and had less frequently a history of a respiratory disease. However, none of these characteristics allow us to identify a subgroup in a reliable way.

## Discussion

### Main findings

In our population-based, cross-sectional sample of adults aged 35–79 years in two northwestern cities in Russia, less than half of those with AO identified during the first spirometry examination had post-BD AO identified during the second spirometry examination. Patients with AO based on both FEV1/FVC cut-off values had mainly been diagnosed with COPD, ACOS, likely ACOS, and asthma with fixed obstruction. A quarter of all the study participants with AO did not report any chronic respiratory symptoms. The PPV of all the respiratory symptoms for the main obstructive (COPD, asthma, ACOS) and non-obstructive (chronic bronchitis) respiratory diseases was low.

This study had three essential findings. First, there was a gap between the “epidemiological” definition (as shown in the baseline RESPECT study) and the “clinical” definition of COPD (as shown in the diagnostic follow-up of the RESPECT study). Second, variability in spirometric values (due to biological and/or measurement error), including pre-BD and post-BD values, was identified. Third, one spirometric assessment was not enough for COPD diagnosis. Serial longitudinal spirometric assessments are needed and should be complemented by a comprehensive clinical assessment when diagnosing COPD.

### Comparison with other studies

#### The difference between epidemiological definitions based on spirometry only and clinical definitions considering symptoms and risk factors

In the majority of previous epidemiological studies, COPD diagnosis has been based on spirometry and a few clinical characteristics, e.g., the absence of self-reported respiratory disease, such as asthma.^4^ One of the main pivotal differences between the epidemiological and clinical diagnosis of COPD is a doctor’s evaluation of risk factors (e.g., smoking history, environmental and occupational hazards), symptoms, and spirometry.^4^ Clinical signs and symptoms, such as dyspnea, cough and mucus production, often vary widely on an individual basis among patients with the same degree of AO.^18^ Systemic inflammation and other clinical manifestations associated with AO may not be captured by FEV1 changes over time but are likely to impact an individual’s clinical severity.^18^

Spirometric evaluation itself may be diagnostic only when values distant from normality are observed.^19^ The diagnosis of COPD is not difficult to assign when values of FEV1/FVC and FEV1 are far from normal, and a patient has respiratory signs and symptoms and known risks factors.^19^ The key dilemma for clinicians is COPD diagnosis when these values are only slightly below the be predicted values (for example, the “gray zone” for FEV1/FVC = 0.70–0.80). The following scenarios have previously been considered.^19^ To confirm the diagnosis of COPD in a subject who has never smoked and has no symptoms, the clinician needs a “no doubt spirometry” result showing AO in a subject who has been exposed to occupational hazards or is an athlete with a high level of cardiovascular and neuromuscular fitness.^19^ If the subject is a current smoker with a history of 70 pack-years and reports chronic cough, phlegm and breathlessness upon moderate effort and has no evidence of chronic heart disease, a more accurate measurement of lung volumes using plethysmography and diffusion capacity is needed to rule out the diagnosis of COPD when FEV1/FVC > 0.70. In this case, it has been suggested to use “clinical” criterion rather than another cut-off value (FEV1/FVC below LLN).^19^

Our study exemplified the differences between epidemiological and clinical perspectives. Of all the participants diagnosed with AO during the first spirometry examination, only 60.8% had evidence of AO observed during the second post-BD spirometry examination. These participants were older (59.5 ± 6.6. years and 56.3 ± 8.8 years, respectively), and had a lower ratio FEV1/FVC observed during the baseline spirometry assessment than did those without AO observed during the second assessment (FEV1/FVC = 0.60 and FEV1/FVC = 0.66, respectively). Of the 62 participants with post-BD AO observed at both the baseline and the second spirometry measurements, 41 (66.1%) had a clinically confirmed diagnosis of COPD, and 13 patients (20.9%) had been diagnosed with ACOS and likely ACOS.

#### The difference between using pre-BD or post-BD spirometry in epidemiological studies

Post-BD spirometric values and the LLN definition have been recommended as the diagnostic criteria to be used when defining COPD in epidemiological studies.^4^ Nevertheless, only pre-BD spirometry testing has been used in some recently published studies.^20^ However, in some other studies, significant differences in COPD prevalence were identified when COPD was diagnosed based on pre-BD and post-BD spirometry measurements.^21,22^ As was demonstrated in the National Health and Nutrition Examination Survey 2007–2010, COPD prevalence among adults aged 40–79 years based on pre-BD measurements was 20.9%, whereas the prevalence based on post-BD measurements was 14.0%.^21^ Similar differences were identified when using the LLN criteria (pre-BD prevalence was 15.4% and post-BD one was 10.2%).^21^ The results of a community-based health checkup study (the Hisayama Study) also supported the aforementioned findings (COPD prevalence rate based on pre-BD measurements was 14.6% in males and 13.7% in females and the rate based on post-BD measurements was 8.7 and 8.7%, respectively).^22^ The authors concluded that the use of post-BD spirometry in health checkups “would reduce the number of subjects with probable COPD by one-third”.^22^

This difference was also demonstrated in the previously published results of the RESPECT study.^17^ In that study, the pre-BD and post-BD AO prevalence rates were 8.3 and 6.8% according to the fixed cut-off value and 5.9 and 4.8% according to GLI–LLN cut-off value, respectively.^17^ In the current study, it is demonstrated that the prevalence rates of pre-BD and post-BD AO changed over time, even without intervention.

#### Difference between using only one spirometry measure and repeated measures for COPD diagnosis

In epidemiological studies, attention has mainly focused on cross-sectional assessments and the influence of COPD criteria on population prevalence; however, the consistency of COPD diagnosis is no less important for patients than are clear spirometric criteria.^13^ There are known and expected variations in spirometric results after repeated testing (due to biological variability and/or measurement errors).^23^ The aforementioned variations and systematic changes due to aging may result in changes in the diagnosis of COPD over time.^13,24^ Based on the GOLD criterion,^2^ a person with an FEV1/FVC = 0.69 would be diagnosed with COPD; however, if the same person had a ratio of 0.7 obtained during a follow-up exam, he/she would no longer be diagnosed as having COPD.^13^ However, the current recommendation for COPD diagnosis only takes into account a single spirometry measurement,^2^ and does not take longitudinal inconsistencies into consideration.^13^

The recently published results of baseline and follow-up studies conducted as part of a multicenter prevalence survey of COPD in major Latin American cities (the PLATINO study) have described the rates and correlates of inconsistent interpretations of AO according to its several criteria.^13^ The follow-up studies were conducted 5–9 years after the baseline surveys were performed.^13^ The following three main findings may be derived from these studies: (1) regardless of which AO criterion was used, inconsistencies in COPD diagnosis were observed (the LLN definition or defined as FEV1/FVC < 0.7 using the GOLD criteria plus FEV1 < 80% of the value predicted for GOLD stages 2–4 or FEV1 < LLN); (2) depending on which AO criterion was used, COPD prevalence would be lower or higher and, therefore, less or more inconsistent upon repeated testing (the FEV1/FVC definition was less concordant when tested twice than was the FEV1/FEV6 definition, while the consistency of COPD diagnosis was highest when using the FEV1/FEV6<LLN definition and for definitions of airflow obstruction requiring a low FEV1 (GOLD stage 2–4); and (3) the closer the FEV1/FVC, FEV1/FEV6 and FEV1 were to the cut points, the higher the possibility of a change in diagnosis upon repeated testing.^13^

The interval between the baseline and follow-up spirometry measurements was shorter in our study than in the aforementioned study (14.5 ± 4.8 months and 5–9 years, respectively). No significant deterioration in health status was observed during this time interval.

In our study, 23.1% of those who did not have post-BD AO observed during the first spirometry were identified as having post-BD AO during the second spirometry measurement. Those who did not have post-BD AO observed during the first assessment but had AO identified at follow-up and those who maintained a non-obstructed airway had similar smoking statuses (86.7 and 66.0% current/ex-smokers, respectively, *p* = 0.24). Similar results were derived among those who had post-BD AO observed at baseline regardless of the follow-up spirometry results (82.3% current/ex-smokers among those with AO at both timepoints and 69.2% among those who did not AO observed at follow-up, *p* = 0.2).

We observed that participants who had AO observed at baseline and during the follow-up spirometry measurement presented a lower ratio FEV1/FVC during baseline spirometry than those who had “reversed” AO.

Of the participants with post-BD obstruction observed during the follow-up spirometry measurement, 47% did not report a diagnosis of any chronic respiratory disease and 26.0% did not report any chronic respiratory symptoms.

Thus, the use of serial longitudinal spirometric assessments seems to be an essential factor in ensuring the stability of COPD diagnoses and should be complemented with comprehensive clinical assessments.

#### Irreversible airway obstruction: COPD, asthma or ACOS?

The GOLD has recently introduced a classification and assessment criteria for different COPD phenotypes, and this introduction has been reflected in several studies employing multidimensional grading systems, such as the BODE (BMI, airflow obstruction, dyspnea and exercise capacity) and other systems aiming to “simplify the complex diagnosis” of a COPD patient.^2,18^

It has been increasingly recognized that both asthma and COPD are heterogeneous diseases with substantial inter-individual variability with regards to their clinical expression and disease progression.^15^ However, there remains a need for a clear distinction between COPD and the irreversible form of asthma. Before the publication of the consensus statement by Global Initiative for Asthma (GINA) and GOLD concerning the diagnosis, assessment, and treatment of ACOS,^25^ there was no clear definition for this overlapping syndrome.^26^ For example, irreversible obstruction in long-smokers was called never-smoker COPD, despite being mainly attributable to asthma.^27^ Another common belief was that COPD and irreversible asthma in smokers could not be differentiated.^28^ In addition, the division of the ACOS phenotype into the two following clinical sub-phenotypes was suggested: (1) never-smokers, ex-smokers, or current smokers with a history of asthma who have incompletely reversible AO (asthma–ACOS); and (2) smokers or ex-smokers with COPD diagnosed according to the GOLD criteria who display increased bronchodilator reversibility or bronchial hyperresponsiveness (COPD–ACOS).^15^

The prevalence of ACOS varies by geographic region and clinical setting (primary or specialist care), and is believed to be high, partially because of the lack of a consistent diagnosis.^29^ It has been estimated that ACOS is present in 15–45% of the population with obstructive airway disease and is believed that the prevalence of ACOS increases with age.^30–32^ Patients with ACOS tend to be older than those with asthma; additionally, they often have a long smoking history, present with asthmatic features, and have persistent AO.^29^ The most recent review article published in the *New England Journal of Medicine* stated that it would be “premature to recommend the designation of ACOS as a disease entity in primary and specialist care.”^32^ Thus, it is essential to better characterize patients with and obtain a standardized definition of ACOS, and a further research is one way to achieve these objectives.^32^

In our study, we used two ACOS definitions based on the current guidelines,^25^ as follows: (1) ACOS: symptoms common to both asthma and COPD, post-BD FEV1/FVC < 0.7 or GLI–LLN and post-BD increase in FEV1 > 12% and 400 ml from baseline (marked reversibility); and (2) likely ACOS: symptoms common to both asthma and COPD, post-BD FEV1/FVC < 0.7 or GLI–LLN and post-BD increase in FEV1 > 12% and 200 ml from baseline (reversible AO). The prevalence of both syndromes (ACOS and likely ACOS) was 9.0% among participants who had AO observed during the first spirometry measurement. Patients with ACOS and likely ACOS tended to be older and have persistent AO, and these patients had the lowest FVC and FEV1 measures of all the study participants.

The PPV of chronic respiratory symptoms was rather low for all final diagnoses but the negative predictive value was high (Table 2). Which means that in the absence of symptoms a diagnosis of COPD/likely COPD, asthma, and chronic bronchitis is highly unlikely. In clinical practice assessing symptoms as a first step might be a defensible strategy. But a word of caution is needed here. The assessment of the diagnostic value of signs and symptoms performed in our analysis might be affected by inclusion bias, since the evaluation of those symptoms is part of the final diagnosis (=reference standard)

#### Strengths and Limitations

The RESPECT study is a prospective population-based study of adults aged 35–70 years with the following three components: a cross-sectional, a case-control and a cohort study. This paper reports the findings of the cross-sectional and cohort components of the RESPECT study. All participants with pre-BD and post-BD AO based on both FEV1/FVC cut-off values in the cross-sectional study were included in the follow-up study and examined by an experienced pulmonologist using a standardized comprehensive diagnostic work-up protocol. Spirometry was performed before and after BD administration and complied with the ATS/ERS standards of spirometry quality.

Our study also has some limitations. One limitation is the participants who were lost to follow-up or refused to take part in the second assessment. For the most part, these participants had moved to other places or were unable to undergo spirometry, and nine participants had died. An additional ten participants were excluded due to low-quality measurements obtained during the second assessment.

Due to financial restrictions and a lack of availability in one of the two cities where the RESPECT study is being conducted, some functional respiratory tests, such as body plethysmography and diffusion capacity, and computer tomography scans were not performed. It should, however, be emphasized that the aim of this study was to improve the diagnosis of AO in primary care settings, which are the first institutions at which patients with respiratory symptoms may be seen.

The RESPECT study population differs from the overall population of the northwestern region of Russia in terms of age and sex.^16^ It has more women than the average population in the northwestern region of Russia (68.2 vs. 55.3%) and less current and ex-smokers than the average Russian population (47.8 vs. 53.9%).^16^ This might have led to a decreased amount of participants being identified as having AO than would be expected in the general population.

## Conclusion

Single spirometry or clinical respiratory symptoms alone are not enough for accurate COPD diagnosis. A comprehensive approach including clinical assessment and follow-up spirometry should be taken into consideration for the diagnosis and management of COPD as well as for any screening program or prevalence study conducted in the future.

## Methods

### Study design and population

The RESPECT study is a population-based study that is being conducted as a collaborative effort between the Université Catholique de Louvain (Belgium), North-Western State Medical University (named for I. I. Mechnikov, St. Petersburg, Russia) and Northern State Medical University, Arkhangelsk (Russia). The study was designed to attain a better understanding of the epidemiology of COPD in northwestern Russia. Descriptions of the design of and rationale for the RESPECT study have already been published elsewhere.^16^ Briefly, 15 primary care centers in two northwestern Russian cities (St. Petersburg and Arkhangelsk) were invited to participate in the RESPECT study, and 15 investigators (10 from St. Petersburg and 5 from Arkhangelsk) were recruited. The study population was comprised of patients randomly selected from lists (organized based on territories) provided by the 15 participating centers. Adults aged 35–70 years were selected from each center using a random number generator and invited to participate in the study. Participating sites agreed to recruit a population-based random sample of at least 200 adults who were not institutionalized, were 35–70 years old, and were living in a well-defined administrative area (16). The research investigators administered questionnaires regarding the participants’ background characteristics, including sociodemographic data, smoking status, occupational exposures and respiratory symptoms. All participants were invited to undergo spirometry. The baseline characteristics of the total RESPECT population have already been published elsewhere.^16^

Those with AO based on the fixed and GLI–LLN cut-off values before (pre-BD) and after (post-BD) bronchodilator administration were invited to participate in this diagnostic study, which included undergoing examination by one of the two principal investigators, who are both experienced pulmonologists, and pre-BD and post-BD spirometry measurement.

For the baseline study, patients were enrolled between June 8, 2012 and December 17, 2013, and those who participated in the diagnostic study were examined between May 16, 2013 and May 13, 2015.

The local medical ethics review boards approved the study protocol (North-West State Medical University [named for I. I. Mechnikov], St. Petersburg, protocol N 11 from 07.12.2011, and Northern State Medical University, Arkhangelsk, protocol N 01/1-12 from 11.01.2012). All participants provided informed consent. Clinical trial registration: NCT02307799. Methods were performed in accordance with relevant regulations and guidelines.

### Background characteristics and other variables

The background characteristics evaluated included sex, age and socioeconomic status. Smoking status was specified as “never smoked”, “ex-smoker” (persons who had quit smoking ≥6 months prior), or “current smoker”. Former and current smokers were asked to report the age at which they began to smoke, how many years they had smoked and how many cigarettes per day they had smoked (in pack-years). One pack-year of smoking indicated that an individual smoked one pack of cigarettes (20 cigarettes) daily for one year. The American Thoracic Society (ATS) 1978 Adult Questionnaire (ATS-DLD-78) was used to assess exposure to occupational hazards.^33^ The participants were asked if they had worked for one year or more in any dusty job, a job with exposure to gas or chemical fumes, and a job involving the use of protective equipment. Information regarding any personal or family history of obstructive airway disease (asthma, chronic bronchitis, emphysema, and chronic cough), allergic diseases, or tuberculosis and the presence of co-morbidities were collected systematically. History of hospitalization, treatment and exacerbation frequency of obstructive disease were assessed at enrollment.

The comprehensive standardized assessment protocol used by the pulmonologist included a physical examination with lung and heart auscultation; measurement of height, weight, BMI, waist circumference, pulse, respiratory rate, and blood pressure; and skin and edema assessments. Patients were asked about the presence of chronic respiratory symptoms, including chronic cough, sputum production (defined as lasting longer than 3 months), and dyspnea.^34^ A 3-level version of the EuroQol 5-Dimension descriptive system (the EQ-5D-3 L) was used as a standardized measure of health status.^35,36^

### Spirometry

Spirometry was performed using a portable turbine Micro Spirometer (MIR Spirobank, Rome, Italy) and a personal computer equipped with the WiPam program to facilitate the uploading of data to a central database. The accuracy of the spirometry measurements that were performed by trained investigators has been previously reported.^37^ All investigators were invited to participate in a 3-week distance-learning course on spirometry with a 1-day face-to-face training session (SpiroCourse).^38^ All investigators completed the course successfully and agreed to receive continuous quality feedback on the performed tests.

Winspiro Pro software (MIR) was used to compare the measured values with those in reference tables and to automatically calculate the reproducibility of the performed spirometry in accordance with the ERS guidelines. Both during the baseline assessment and this diagnostic study, pre-BD and post-BD spirometry were performed using 400 μg of salbutamol or 160 μg of ipratropium bromide (for the patients older than 60 years of age or with comorbid cardiovascular disease). The ATS/ERS quality criteria were used to assess the acceptability and repeatability of the results.^23^ All spirograms were evaluated by two independent experts and classified into 4 categories (ATS1: all ATS/ERS criteria, including reproducibility, are fulfilled; ATS2: all criteria except for duration of expiration >6 s are fulfilled; ATS3: the test was “usable” for the interpretation of the peak expiratory flow and FEV_1_, and the spirograms displayed good starts and no coughs during the 1st second of the maneuver; and ATS4: none of the ATS/ERS criteria are fulfilled and spirograms are not usable). Spirograms classified as ATS1 or ATS2 were considered to be of acceptable quality for inclusion in this study.

The predictive values of the spirometry parameters were calculated based on the GLI 2012 reference values using GLI 2012 Data Conversion software.^39^ Pre-BD and post-BD AO was defined using FEV_1_/FVC < 0.7 (fixed cut-off) and FEV_1_/FVC<GLI–LLN as cut-off values.

### Statistical analysis

Descriptive statistics were calculated for all variables. Continuous variables are presented as the mean ± standard deviation (SD), and categorical variables are presented as numbers with frequencies.

After baseline spirometry was performed, the study population was divided into the following two main AO categories: (1) participants with pre-BD AO (FEV1/FVC < 0.7 and/or FEV1/FVC < GLI–LLN), and (2) participants with post-BD AO (FEV1/FVC < 0.7 and/or FEV1/FVC<GLI–LLN). After the second spirometry with BD test, both categories were further divided into the following subgroups: (a) participants with post-BD AO (FEV1/FVC < 0.7 and/or FEV1/FVC<GLI–LLN); and (b) participants without AO according to either cut-off value (FEV1/FVC ≥ 0.70 or FEV1/FVC ≥ GLI–LLN).

**Fig. 2:**
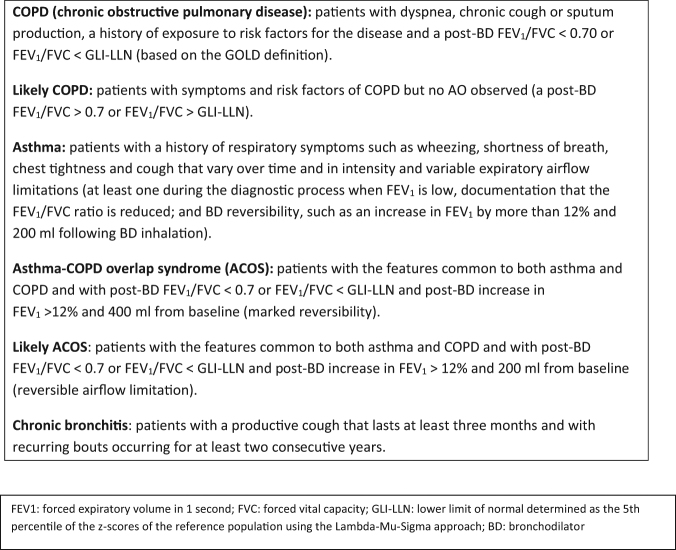
Diagnostic criteria box for patients diagnosed with respiratory diseases

With reference to clinical and spirometric criteria, participants were assigned to one of the following mutually exclusive diagnostic categories: (1) COPD, (2) likely COPD, (3) asthma, (4) ACOS, (5) likely ACOS, (6) chronic bronchitis, (7) other diagnosis and (8) no objective evidence of obstructive lung disease (Fig. 1). The diagnosis of obstructive disease was based on the guidelines of GOLD (2), the GINA^40^ and the diagnosis of diseases of chronic airflow limitation: asthma, COPD and ACOS^25^ (Fig. 2).

Statistical significance was set at <0.05 (a two-tailed probability value). SPSS version 20.0 (SPSS Inc., Chicago, IL, USA) was used for the statistical analyses.

### Data availability

The datasets generated and/or analyzed during the current study are available from the corresponding author on reasonable request.

## Electronic supplementary material

Supplementary Table 1

Supplementary Table 2
