# Supplementary material for: Spirometry is not enough to diagnose COPD in epidemiological studies: a follow-up study
Source: NPJ Prim Care Respir Med. 2017 Nov 14;27:62. doi: 10.1038/s41533-017-0062-6 (PMC5686137; doi:10.1038/s41533-017-0062-6)
Supplement: Supplementary file 1 — Supplementary Table 1 [file 41533_2017_62_MOESM1_ESM.pdf]

Supplementary table 1. Comparison of the shift in diagnosis from obstructive to non-obstructive after the final assessment, in four different groups of patients, stratified by their *baseline* GOLD stage label.

| | Total number of participants with a diagnosis of AO at baseline (n= 102) | Still obstructive after the second assessment (n= 62) | Non-obstructive after the second assessment (n = 40) |
|---|---|---|---|
| GOLD 1 ($FEV_1 \geq 80\%$) | 43 (42.2%) | 19 (30.6%) | 24 (60%) |
| GOLD 2 ($50\% \leq FEV_1 < 80\%$) | 50 (49.0%) | 35 (56.5%) | 15 (37.5%) |
| GOLD 3 ($30\% \leq FEV_1 < 50\%$) | 8 (7.8%) | 7 (11.3%) | 1 (2.5%) |
| GOLD 4 ($FEV_1 < 30\%$) | 1 (2.0%) | 1 (1.6%) | 0 |

GOLD - the Global Strategy for the Diagnosis, Management and Prevention of COPD, Global Initiative for Chronic Obstructive Lung Disease
$FEV_1$ - Forced expiratory volume in 1 s
