# Supplementary material for: Spirometry is not enough to diagnose COPD in epidemiological studies: a follow-up study
Source: NPJ Prim Care Respir Med. 2017 Nov 14;27:62. doi: 10.1038/s41533-017-0062-6 (PMC5686137; doi:10.1038/s41533-017-0062-6)
Supplement: Supplementary file 2 — Supplementary Table 2 [file 41533_2017_62_MOESM2_ESM.pdf]

Supplementary table 2. Baseline characteristics of the participants with and without a diagnostic shift after the second assessment (total n = 102)

	Those who were confirmed to be obstructive during the second assessment (n= 62)	Those who shifted to non-obstructive after the second assessment (n = 40)	P value
Age, mean ± SD	59.5 ± 6.6	56.4 ± 8.8	**<0.05***
Men, %	38 (61.3%)	20 (50%)	0.36
Smokers (ex-smokers), %	51 (82.3%)	28 (70%)	0.23
Smoking exposure [a], n (%)			
Never smoking	11 (17.7%)	13 (32.5%)	0.14
< 10 pack-years	10 (16.1%)	6 (15%)	0.90
≥10 and < 20 pack-years	5 (8.1%)	5 (12.5%)	0.70
Smoking 20+ pack/years	36 (58.1%)	16 (40%)	0.11
Dusty job, ≥ 10 years, n (%)	14 (23.7%)	13 (34.2%)	0.31
Gas (fumes) job ≥ 10 years, n (%)	17 (28.8%)	13 (34.2%)	0,.32
Symptoms at the baseline study			
Any cough, n (%)	36 (58.1%)	23 (59%)	0.91
Any dyspnea, n (%)	37 (59.7%)	21 (53.8%)	0.71
All 3 chronic respiratory symptoms together [b], n (%)	20 (32.3%)	7 (17.9%)	0,17
Family history of asthma, n (%)	3 (4.8%)	6 (15.4%)	0.14
FEV1 (L), post BD baseline, mean ± SD	2.19 ± 0.72	2.56 ± 0.72	**<0.05***
FVC (L), post BD baseline, mean ± SD	3.62 ± 1.04	3.84 ± 1.00	0.29
FEV1/FVC, post BD baseline, mean ± SD	60.3 ± 9.3	66.3 ± 3.40	**<0.01***
BMI, kg/m2, mean ± SD	26.9 ± 5.1	26.8 ± 5.7	0.93
Current respiratory disease (as reported by the patient)			
Asthma, n (%)	16 (26.2%)	4 (10.3%)	0.09

Any allergic diseases, n (%)	13 (23.3%)	10 (25.6%)	0.80
Chronic bronchitis, n (%)	21 (34.4%)	3 (7.7%)	**<0.01****
COPD, n (%)	14 (23%)	0 (0%)	**<0.01****
Any respiratory diseases, n (%)	34 (55.7%)	6 (15.4%)	**<0.01****

Figures are means ± SD unless indicated otherwise.
[a] One pack-year of smoking indicates that an individual smoked one package of cigarettes (20 cigarettes) daily for 1 year.
[b] Chronic respiratory symptoms were defined as the presence of chronic cough and chronic phlegm (on most days for as long as 3 months each year), together with chronic dyspnea.
FEV_1 - Forced expiratory volume in 1 s
FVC - Forced vital capacity
FEV_1/FVC – a ratio of the forced expiratory volume in 1 s to the forced vital capacity
BMI – Body mass index
COPD – Chronic obstructive pulmonary disease
*Significant differences (P < .05) within the shift group (p-value for one sample t-test)
** Significant differences (P < .05) within the shift group (p-value for Pearson Chi-square test)
